# The Hypothesis of Trace Elements Involvement in the Coronary Arteries Atherosclerotic Plaques’ Location

**DOI:** 10.3390/jcm13226933

**Published:** 2024-11-18

**Authors:** Tomasz Urbanowicz, Anetta Hanć, Julia Frąckowiak, Jakub Piecek, Ievgen Spasenenko, Anna Olasińska-Wiśniewska, Beata Krasińska, Andrzej Tykarski

**Affiliations:** 1 Cardiac Surgery and Transplantology Department, Poznan University of Medical Sciences, 61-701 Poznan, Poland; 2 Department of Trace Analysis, Faculty of Chemistry, Adam Mickiewicz University, 61-614 Poznan, Poland; 3 Scientific Students’ Group, Poznan University of Medical Sciences, 61-701 Poznan, Poland; 4 Department of Hypertensiology, Angiology and Internal Medicine, Poznan University of Medical Sciences, 61-701 Poznan, Poland

**Keywords:** trace elements, coronary disease, scalp hair, atherosclerosis, minerals, LAD, Cx, RCA

## Abstract

**Background**: Coronary artery disease (CAD) is a chronic inflammatory disease with multiple well-known risk factors. Although epidemiological studies report improvements in classical CAD risk-factor control, except for diabetes and obesity, cardiovascular diseases remain the leading causes of morbidity and mortality in the current population. The question regarding the atherosclerotic plaque location in particular arteries remains unanswered. Research on novel possible aspects that could help to properly understand atherosclerosis pathophysiology is essential. This study was based on a body trace-elements analysis, measured in scalp hair samples, as possible co-factors of various enzymes that may be crucial for CAD development. **Methods**: A total of 133 consecutive male patients with a median age of 71 (65–75) years, who presented with anginal symptoms of CCS class 2.0 (0.3) without previous heart-related interventions, were included in the analysis. The results of the cine-angiography were compared with the demographical, clinical, and laboratory results, followed by scalp-hair trace analysis. The possible predictors for coronary disease locations in the left descending artery (LAD), the circumflex artery (Cx), and the right coronary artery (RCA) were the subjects of this study. **Results**: Statistically significant differences in the scalp-hair trace elements concentration between the CAD and normal angiogram groups were noticed for magnesium (*p* = 0.003), calcium (*p* < 0.001), chromium (*p* = 0.011), and copper (*p* = 0.043). The multivariable analysis for epicardial atherosclerotic disease revealed the co-existence of diabetes mellitus (OR: 2.94, 95% CI: 1.27–6.79, *p* = 0.012) as a possible causative factor for the LAD location. The multivariable analysis for the atherosclerosis location in the Cx artery presented scalp-hair magnesium as a possible predictive factor (OR: 0.98, 95% CI: 0.96–1.00, *p* = 0.024). The multivariable model for the RCA location of atherosclerotic plaque indicated scalp-hair Zn concentration (0.99, 95% CI: 0.98–1.00, *p* = 0.002) and serum HDL (OR: 0.61, 95% CI: 0.04–0.09, *p* = 0.016). **Conclusions**: Possible hypothetical distinctive pathomechanisms, in particular, coronary artery involvement, in atherosclerosis processes are presented in the male group. Diabetes mellitus was found to be the primary factor for left descending artery disease. The low scalp-hair magnesium concentration was found to be a possible risk factor involved in the circumflex artery atherosclerotic plaque location. The inverse relation between serum high-density lipoprotein, the scalp hair zinc concentration, and right coronary disease was noticed.

## 1. Introduction

Coronary artery disease (CAD) is a current clinical challenge [1]. It belongs to multifactorial complex diseases characterized by underlying manifold pathomechanisms. In addition to traditional risk factors, its metabolomic profile is distinctive from healthy controls [2]. Age, sex, genetic predisposition, arterial hypertension, dyslipidemia, sedentary lifestyle, and nicotine and alcohol intake have been linked to the disease [3]. Certain pathways that are involved in energy and lipoprotein metabolism, inflammatory activation, and antioxidant hemostasis derangements, in addition to impaired DNA damage repair, are among possible factors on the molecular level [4,5,6].

The vast majority of patients with diagnosed atherosclerotic disease are males [7]. The genetic, clinical, and lifestyle-related risk factors are well-described [8]. Preventive therapy has succeeded, as most co-morbidities are well-controlled nowadays [9], though the number of patients presenting with CAD diagnoses remains stable. Further investigation is still necessary to highlight additional factors that could be modified from an epidemiological perspective. Recent papers pointed out that metabolic deviations in CAD patients are person-specific and related to genetic or environmental bases [2]. CAD is regarded as a chronic inflammatory pathology. As trace elements are regarded as enzymatic co-factors, our recent study highlighted the possible correlation between the CAD location and scalp hair trace-elements concentration [10].

There is still a gap in our knowledge related to the pathophysiology of atherosclerotic locations, in particular for the coronary arteries. Little is known about why specific arteries are involved in atherosclerosis formation. The rheological implication of atherosclerotic plaque formation via wall-stress shear is postulated [11], but the primary question of the origin of the disease in particular coronary arteries has not been answered.

This study aimed to examine scalp hair samples in male patients presenting with chronic coronary syndrome and to compare them with the results of the cine-angiography.

## 2. Materials and Methods

### 2.1. Patients

A total of 133 male patients with a median age of 71 (65–75) years who presented with anginal symptoms of the CCS class 2.0 (0.3) without previous heart-related interventions were included in the analysis. All participants were referred for cine-angiography by a cardiologist and hospitalized in the Internal Medicine and the Hypertensiology departments between 2000–2022. The inclusion criteria were limited to the white Caucasian population who presented with standard dietary habits.

The patients’ scrutinized medical histories were collected, and their clinical symptoms were evaluated. Scalp hair samples were taken for chemical examination upon admission. A laboratory test, transthoracic echocardiography, was performed prior to the angiograms. The results obtained were compared with those of the chemical analysis of the scalp hair samples.

Patients were diagnosed with co-existences of arterial hypertension (114 pts (86%)), diabetes mellitus type 2 (46 pts (35%)), and hypercholesterolemia (120 pts (90%)). A total of 67 (50%) patients admitted to nicotine addiction, including 27 (20%) active smokers.

The exclusion criteria included female sex, acute coronary syndromes, and previous coronary revascularization. Patients on restrictive diets and presenting histories of oncological therapy were not included in this study.

### 2.2. Hair Sample Analysis

Hair samples, 2–3 cm long and untreated with perm or dye, were cut from the occipital region of the head, close to the scalp. The collected samples were washed and dried, following the procedure described in [12]. A dry sample weighing 150–200 mg was mineralized using the DigiTUBE system (DigiTUBE Science, Quebec, QC, Canada). A total of 4 mL of 65% nitric acid (Suprapur, Merck, Darmstadt, Germany) and 1 mL of 30% hydrogen peroxide (Supelco, Merck, Darmstadt, Germany) were used for mineralization. The prepared samples were heated at 150 °C for 4 h. After cooling to room temperature, the samples were diluted 100 times with Milli-Q water (Millipore Direct Q-3, Merck, Darmstadt, Germany). These samples were then analyzed for elemental content using the SN-ICP-MS method (7700x Agilent, Santa Clara, CA, USA) described by Urbanowicz et al. [12]. The validity of the analytical method was assessed by analyzing the certified reference material (CRM), NCS ZC 81002b Human Hair (Beijing, China). Trueness was evaluated using the CRM and expressed as recovery values (%) ranging from 94% to 107%.

### 2.3. Statistical Analysis

Because the data did not follow a normal distribution (Shapiro–Wilk test), the parameters were presented as medians and interquartile ranges (Q_1_–Q_3_). The categorical data were presented as numbers and percentages. The comparison between the groups was performed by the Kruskal–Wallis test with post-hoc Dunn’s tests. If the comparison considered categorical data, the chi-square test of independence was used.

Two sample Wilcoxon (Mann–Whitney) tests were performed to present the differences between the measured parameters, including laboratory and clinical results.

The uni- and multivariable models were created for particular coronary artery disease prediction. The results were presented as odds ratios (OR) and 95% confidence intervals (95% CI).

Statistical analysis was performed with the use of JASP Team (2020)^®^ JASP version 0.14.1 (University of Amsterdam, Ostend, The Netherlands; https://www.jasp-stats.org/download/, accessed on 14 October 2020). All tests were considered significant at *p* < 0.05.

### 2.4. Bioethics Committee Approval

This study was performed according to the principles of Good Clinical Practice and the Declaration of Helsinki. It was approved by the Local Ethics Committee of the Medical University of Poznan (approval number: 875/22 on 3 November 2022). All patients gave their informed consents for inclusion in the study.

## 3. Results

Normal angiograms were found in 63 (47%) patients. The mean (SD) number of atherosclerotic lesions was 2.0 (0.9) in 73 (53%) patients. The age (*p* = 0.386) clinical CCS class presentation (*p* = 0.934) and the comorbidities analysis between both groups did not reveal any significant differences, as presented in Table 1. The difference between the previous and active smokers was not distinctive (*p* = 0.790 vs. *p* = 0.713, respectively). The laboratory results did not indicate significant differences in the lipidogram results or the kidney (*p* = 0.141) and liver (*p* = 0.833) tests. The serum uric acid concentrations were not differentiating (*p* = 0.151).

The analyzed groups did not differ regarding the remaining left-ventricular ejection fraction performance in all patients within the normal range. The cine-angiography revealed coronary artery disease requiring percutaneous intervention involving the left main coronary (3 (2%) patients), the left descending artery (45 (34%) patients), and the circumplex artery (27 (20%) pts), followed by right coronary artery (32 (24%)). The detailed results of the cine-angiography are presented in Table 2.

### 3.1. Scalp Hair Analysis

The scalp hair trace-elements analysis was performed in relation to the coronary artery confirmation in the cine-angiographic results presenting statistical differences, as presented in Table 3.

**Figure 1:**
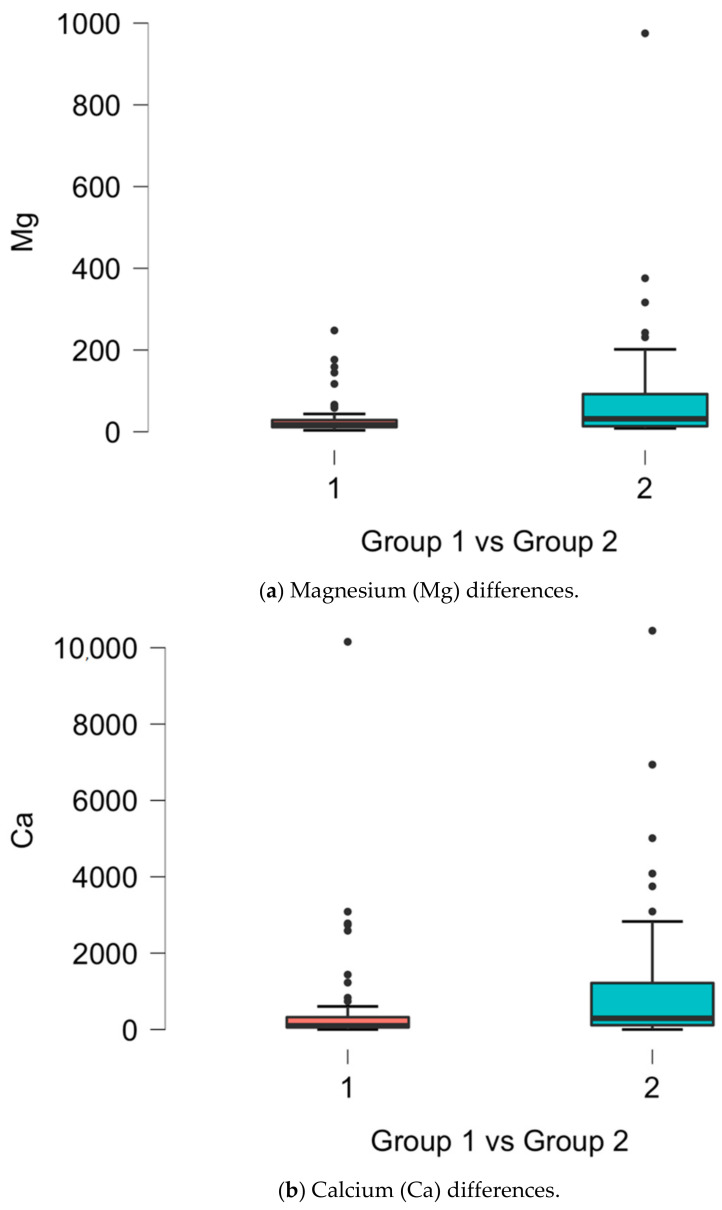

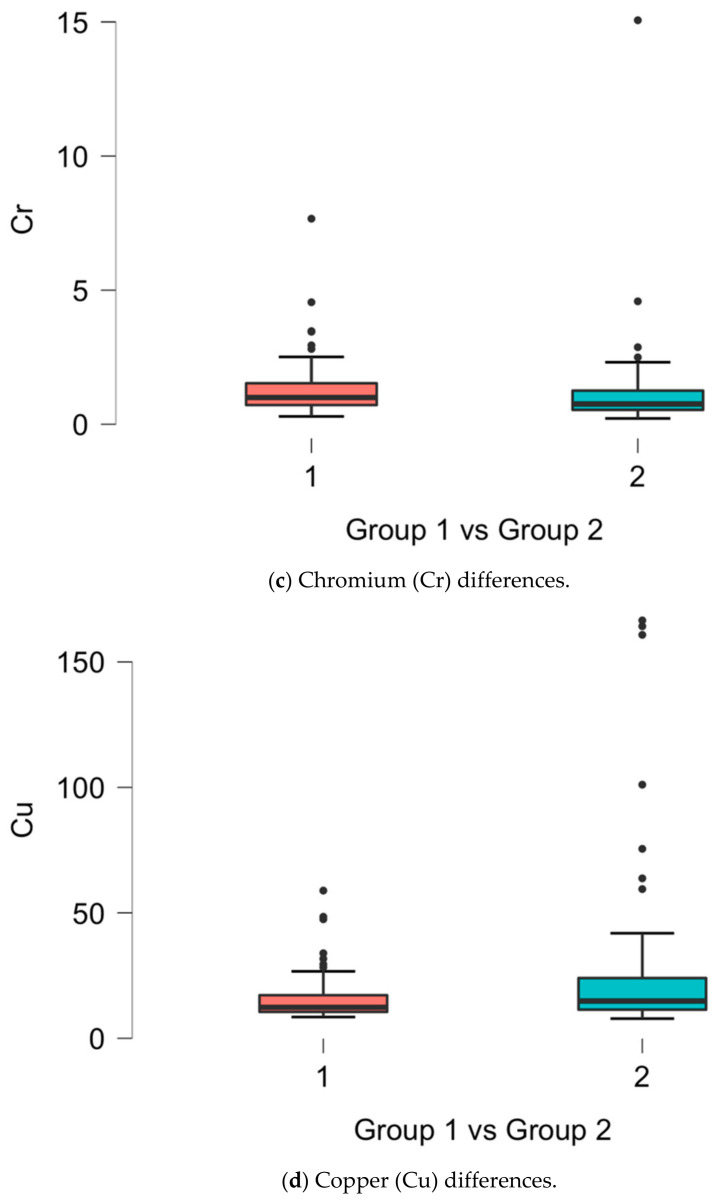
Significant differences in scalp hair trace elements (Mg, Ca, Cr, Cu) between the presented groups (**a**–**d**) [mg/kg].

Statistically significant differences in the scalp hair trace-elements concentration between the CAD and normal angiogram groups were noticed for magnesium (*p* = 0.003), calcium (*p* < 0.001), chromium (*p* = 0.011), and copper (*p* = 0.043), as presented in Figure 1.

### 3.2. Multivariable Models

#### 3.2.1. Multivariable Analysis for Left Descending Artery Disease (LAD) Prediction

The univariable and multivariable models for coronary artery disease in LAD were created as shown in Table 4. The univariable analysis revealed the predictive roles of serum glucose (OR: 1.26, 95% CI: 1.01–1.57, *p* = 0.038) and diabetes mellitus (OR: 2.20, 95% CI: 1.06–4.57, *p* = 0.034) on LAD risk. The multivariable analysis presented DM as a possible predictive factor (OR: 2.94, 95% CI: 1.27–6.79, *p* = 0.012). If created for the following factors, excluding the scalp hair trace-elements concentration, the same multivariable model was inconclusive for any of the predictors.

#### 3.2.2. Multivariable Analysis for Circumflex Artery Disease (Cx) Prediction

The univariable and multivariable models for coronary artery disease in Cx were created as shown in Table 5. The univariable analysis revealed the predictive roles of magnesium (OR: 0.98, 95% CI: 0.96–1.00, *p* = 0.016) and calcium (OR: 0.99, 95% CI: 0.99–1.00, *p* = 0.030). The multivariable analysis also presented magnesium as a possible predictive factor (OR: 0.98, 95% CI: 0.96–1.00, *p* = 0.024). If created for the following factors, excluding the scalp hair trace-elements concentration, the same multivariable model was inconclusive for any of the predictors.

#### 3.2.3. Multivariable Analysis for Right Coronary Artery Disease (RCA) Prediction

The univariable and multivariable models for coronary artery disease in RCA was created as shown in Table 6. In the multivariable model, scalp hair Zn concentration (0.99, 95% CI: 0.98–1.00, *p* = 0.002) and serum HDL (OR: 0.61, 95% CI: 0.04–0.09, *p* = 0.016) were found to be predictive for right coronary artery disease. If created for the following factors, excluding the scalp hair trace-elements concentration, the same multivariable model was inconclusive for any of the predictors.

## 4. Discussion

The results of our analysis point out the differences between atherosclerotic involvements in particular coronary arteries in male patients. In the first step of our analysis, we noticed significant differences between concentrations of magnesium, calcium, copper, and zinc in the scalp hair of the coronary disease and the normal angiogram groups. Apart from copper, we found significantly lower magnesium, calcium, and chromium levels in patients with coronary artery atherosclerosis. The multivariable models for particular coronary artery involvement were created in the second step of the analysis. We hypothesized that there may be differences in the pathophysiological background of atherosclerotic plaque formation in particular coronary arteries. According to our analysis, magnesium and zinc may have a modulatory effect on trace elements regarding atherosclerosis location in the circumflex and right coronary arteries.

Our analysis highlights the significance of traditional risk factors for coronary artery disease development, such as diabetes mellitus and serum high-density lipoprotein.

The predictive role of diabetes on left descending artery disease was noted in our analysis. Diabetes mellitus (DM) is a chronic noncommunicable disease that affects approximately 9.3% of the world’s population, having significant impacts on health and doubling the risk of major cardiovascular events [13]. It is now appreciated that diabetes mellitus and CAD are two chronic disorders that share some common mechanisms, including endothelial and vascular smooth-muscle cell dysfunction, macrophage activation, cytokines upregulation, and metabolic pathway alteration [14]. In hyperglycemia, advanced glycation end-product upregulation and endothelial dysfunction induce a surge of biomolecules, such as vascular endothelial growth factor and plasminogen activator inhibitor-1 (PAI-1), which are essential in atherosclerotic plaque formation [15]. As the left coronary system is more susceptible to atherosclerotic involvement, the strong relationship between DM and CAD from a clinical perspective is highlighted [16]. In previous studies [17], the rheological hypothesis regarding the high predisposition of LAD involvement in atherosclerosis processes was explained by local hemodynamic forces. The wall shear stress, which activates the inflammatory response by blood rheology parameters, may be especially pronounced in diabetic patients, linking DM and LAD location to atherosclerotic plaque development. We may hypothesize that atherosclerotic LAD involvement can be related to wall shear stress and diabetic-related pathomechanisms.

Our analysis noticed the protective role of high-density lipoprotein concentrations on right coronary artery disease. The atheroprotective role of HDL is complex and related to the reversal of cholesterol transport, antioxidant and anti-inflammatory properties, and the ability to maintain endothelial homeostasis [18]. As HDL is a complex molecule, and its properties may vary among the patients, additional factors including nicotine addiction and the co-existence of diabetes mellitus or inflammatory activation are believed to be powerful function modulators [19,20].

Our analysis linked the increased risk for atherosclerosis located in the circumflex artery with a lower scalp hair magnesium concentration. According to the analysis, the inverse relationship between magnesium and atherosclerosis may possibly indicate that magnesium supplementation can be beneficial against plaque formation. The role of magnesium in cardiovascular risk was presented by An et al. [21]. In the Alpha Omega cohort, magnesium supplementation reduced cardiovascular and all-cause mortality [22]. The possible beneficial effect of magnesium supplementation on CAD risk was presented in the Larsson et al. meta-analysis [23]. In their analysis, Veronese et al. [24] presented the effects of Mg intake on inflammatory markers such as the IL-6 levels secreted by T cells and macrophages. Based on our results, we may hypothesize that a lower magnesium concentration may trigger inflammatory activation, resulting in atherosclerosis development in the circumflex artery.

The multivariable model for right coronary artery involvement by atherosclerosis processes pointed out the predictive role of zinc concentration. The anti-inflammatory and antioxidative capabilities of zinc suggest that its deficit may increase the risk of developing cardiovascular diseases (CVDs). Nazari et al. [25] indicated significant changes in interleukin-6 (IL-6), tumor necrosis factor-α (TNF-α), nitric oxide (NO), total antioxidant capacity (TAC), and total glutathione (GSH) related to zinc intake.

We want to indicate a possible distinct mechanism in atherosclerotic plaque formation in relation to particular coronary arteries. Our analysis is based on the concentration of trace elements in the body, as measured by scalp hair analysis. As the trace elements are co-factors for distinct enzymatic processes, the possible mechanisms of atherosclerosis formation, in particular, those of coronary arteries, may vary. The results from our report suggest possible mechanisms that require further analysis but may answer the primary question of why coronary arteries are not equally involved in atherosclerosis formation.

### Study Limitation

This study was performed as a single-center analysis for patients presenting with chronic coronary syndrome in males. As CAD is a multifactorial disease, the presented results may indicate one of the possible explanations of particular coronary artery involvements by atherosclerotic processes. This study presents a hypothesis that needs to be verified on a larger population.

## 5. Conclusions

In this study, possible hypothetical distinctive pathomechanisms in atherosclerosis processes, in particular for coronary artery involvement, were presented in the male group. Diabetes mellitus was found to be the primary factor for left descending artery disease. Low scalp hair magnesium concentration was found to be a possible risk factor involved in circumflex artery atherosclerotic plaque location. The inverse relation between serum high-density lipoprotein, scalp hair zinc concentration, and right coronary disease was noticed.

## Figures and Tables

**Table 1:** Group characteristics.

Parameters	Group 1 CAD Group n = 73	Group 2 Normal Angiograms n = 63	*p*
Demographical			
Age (years) (median (Q_1_–Q_3_))	71 (65–74)	70 (64–75)	0.386
Clinical:			
CCS (class) (mean (SD))	1.9 (0.4)	2.0 (0.2)	0.945
Co-morbidities			
Dyslipidemia (n (%))	69 (95)	51 (81)	0.067
Arterial hypertension (n (%))	66 (90)	48 (37)	0.09
Diabetes mellitus (n (%))	22 (30)	24 (38)	0.367
Nicotine:			
all (n (%))	38 (52)	29 (46)	0.672
active (n (%))	16 (22)	11 (18)	0.613
past (n (%))	22 (30)	18 (29)	0.79
Laboratory:			
WBC (10 × 9/dL) (median (Q_1_–Q_3_))	7.45 (6.35–9.07)	6.73 (5.54–8.77)	0.115
Hemoglobin (mmol/dL) (median (Q_1_–Q_3_))	8.8 (7.9–9.3)	8.5 (8.0–9.0)	0.446
Platelets (10 × 3/dL) (median (Q_1_–Q_3_))	220 (173–258)	211 (184–242)	0.741
ALT (I.U./dL) (median (Q_1_–Q_3_))	25 (17–35)	24 (18–37)	0.833
Creatinine (umol/dL) (median (Q_1_–Q_3_))	85 (77–101)	82 (75–93)	0.147
Serum glucose (mmol/L) (median (Q_1_–Q_3_))	6.1 (5.5–7.7)	5.9 (5.4–5.8)	0.418
Total cholesterol (mmol/L) (median (Q_1_–Q_3_))	3.7 (3.2–4.7)	4.0 (3.4–5.0)	0.291
LDL (mmol/L) (median (Q_1_–Q_3_))	1.93 (1.40–2.90)	2.38 (1.70–2.73)	0.337
HDL (mmol/L) (median (Q_1_–Q_3_))	1.21 (0.97–1.47)	1.24 (1.09–1.56)	0.139
Triglycerides (mmol/L) (median (Q_1_–Q_3_))	1.34 (1.07–1.76)	1.30 (0.99–1.65)	0.377

Abbreviations: ALT—alanine transaminase, CCS—Canadian Cardiology Society, dL—deciliter, HDL—high-density lipoprotein, LDL—low-density lipoprotein, mmol—millimole, n—number, umol—micromole, SD—standard deviation, Q—quartile, and WBC—white blood count.

**Table 2:** Cine-angiography results in the presenting groups.

Parameters	Group 1 CAD Group n = 73	Group 2 Normal Angiograms n = 63	*p* (Group 1 vs. 2)
Cine-angiography			
LMCA			
normal (n (%))/significant stenosis (n (%))	70 (96)/3 (4)	63 (100)/0 (0)	0.249
LAD			
normal (n (%))/significant stenosis	28 (38)/45 (62)	63 (100)/0 (0)	<0.001
Cx			
normal (n (%))/significant stenosis(n (%))	46 (63)/27 (37)	63 (100)/(0)	<0.001
RCA			
normal (n (%))/significant stenosis (n (%))	41 (56)/32 (44)	63 (100)/(0)	<0.001
Echocardiography:			
LVEF (%) (median (Q_1_–Q_3_))	60 (55–63)	58 (55-67)	0.834

Abbreviations: Cx—circumflex artery, LAD—left descending artery, LMCA—left main, LVEF—left ventricular ejection fraction, n—number, RCA—right coronary artery, and Q—quartile. Significant—defined as at least 50% lumen stenosis.

**Table 3:** Scalp hair trace-elements concentration in relation to coronary artery disease.

Trace Elements Concentration	Group 1 CAD Group n = 73	Group 2 Normal Angiograms n = 63	*p*
Mg concetration (mg/kg) (median (Q_1_–Q_3_))	31.747 (13.463–92.158)	17.241 (11.202–28.684)	0.003
Ca concetration (mg/kg) (median (Q_1_–Q_3_))	293 (111.263–1217.154)	100.4 (54.772–322.712)	<0.001
Cr concetration (mg/kg) (median (Q_1_–Q_3_))	0.756 (0.537–1.255)	0.999 (0.717–1.529)	0.011
Fe concetration (mg/kg) (median (Q_1_–Q_3_))	10.254 (8.625–13.007)	11.539 (8.746–15.443)	0.129
Cu concetration (mg/kg) (median (Q_1_–Q_3_))	14.852 (11.405–24.012)	12.360 (10.518–17.163)	0.043
Zn concetration (mg/kg) (median (Q_1_–Q_3_))	157.029 (126.970–172.237)	148.872 (116.407–168.705)	0.394

Abbreviations: Ca—calcium, CAD—coronary artery disease, Cr—chromium, Cu—copper, Fe—iron, kg—kilogram, Mg—magnesium, mg—milligrams, n—number, Q—quartile, and Zn—zinc.

**Table 4:** Uni- and multivariable models for LAD disease prediction in male patients.

Parameters	Univariable			Multivariable		
	OR	95% CI	*p*	OR	95% CI	*p*
Age	1.04	1.00–1.09	0.057			
Clinical:						
HA	1.33	0.50–3.54	0.572			
DM	2.2	1.06–4.57	0.034	2.94	1.27–6.79	0.012
Dyslipidemia	1.55	0.48–5.00	0.532			
Nicotine (all)	1.4	0.71–2.76	0.339			
Laboratory:						
HDL	0.94	0.39–2.27	0.892			
LDL	1.02	0.96–1.08	0.575			
TG	1.16	0.79–1.72	0.446			
creatinine	1	0.98–1.01	0.722			
serum uric acid	1	1.00–1.01	0.225			
serum glucose	1.26	1.01–1.57	0.038			
Trace elements:						
Mg	1	0.99–1.00	0.12			
Ca	1	1.00–1.00	0.275			
Cr	1.04	0.83–1.29	0.756			
Fe	1	0.99–1.01	0.586			
Cu	0.99	0.98–1.01	0.31			
Zn	1	0.99–1.00	0.302			

Abbreviations: Ca—calcium, Cr—chromium, Cu—copper, CI—confidence interval, DM—diabetes mellitus, Fe—iron, HA—arterial hypertension, HDL—high-density lipoprotein, LDL—low-density lipoprotein, Mg—magnesium, OR—Odds ratio, TG—triglycerides, and Zn—zinc.

**Table 5:** Uni- and multivariable models for Cx disease prediction in male patients.

Parameters	Univariable			Multivariable		
	OR	95% CI	*p*	OR	95% CI	*p*
Age	1.33	0.99–1.08	0.186			
Clinical:						
HA	1.66	0.51–5.37	0.396			
DM	1.09	0.50–2.37	0.838			
Dyslipidemia	5.56	0.70–44.33	0.105			
Nicotine (all)	0.68	0.32–1.44	0.314			
Laboratory:						
HDL	1.16	0.45–3.03	0.755			
LDL	1.02	0.96–1.09	0.531			
TG	0.67	0.36–1.26	0.213			
creatinine	1	0.98–1.01	0.551			
serum uric acid	1	1.00–1.00	0.825			
serum glucose	1.02	0.83–1.25	0.869			
Trace elements:						
Mg	0.98	0.96–1.00	0.016	0.98	0.96–1.00	0.024
Ca	0.99	0.99–1.00	0.03			
Cr	1.07	0.85–1.34	0.575			
Fe	1	0.99–1.01	0.683			
Cu	0.98	0.95–1.01	0.18			
Zn	1	0.99–1.01	0.58			

Abbreviations: Ca—calcium, Cr—chromium, Cu—copper, CI—confidence interval, DM—diabetes mellitus, Fe—iron, HA—arterial hypertension, HDL—high-density lipoprotein, LDL—low-density lipoprotein, Mg—magnesium, OR—Odds ratio, TG—triglycerides, and Zn—zinc.

**Table 6:** Uni- and multivariable models for RCA disease prediction in male patients.

Parameters	Univariable			Multivariable		
	OR	95% CI	*p*	OR	95% CI	*p*
Age	1.07	1.02–1.12	0.007			
Clinical:						
HA	1.08	0.39–2.95	0.884			
DM	0.79	0.38–1.67	0.539			
Dyslipidemia	2.22	0.58–8.49	0.243			
Nicotine (all)	0.62	0.31–1.26	0.189			
Laboratory:						
HDL	0.64	0.43–0.94	0.024	0.61	0.04–0.91	0.016
LDL	1.07	0.34–2.63	0.884			
TG	0.66	0.37–1.18	0.159			
creatinine	1	0.98–1.01	0.919			
serum uric acid	1	0.99–1.00	0.309			
serum glucose	1.05	0.86–1.27	0.656			
Trace elements:						
Mg	0.99	0.99–1.00	0.084			
Ca	1	1.00–1.00	0.36			
Cr	0.83	0.59–1.17	0.295			
Fe	0.96	0.91–1.02	0.179			
Cu	0.98	0.96–1.01	0.148			
Zn	0.99	0.98–1.00	0.004	0.99	0.98–1.00	0.003

Abbreviations: Ca—calcium, Cr—chromium, Cu—copper, CI—confidence interval, DM—diabetes mellitus, Fe—iron, HA—arterial hypertension, HDL—high-density lipoprotein, LDL—low-density lipoprotein, Mg—magnesium, OR—Odds ratio, TG—triglycerides, and Zn—zinc.

## Data Availability

The created data and analysis will be available for three years following the publication of this article upon reasonable request to the corresponding author.
